# Assessment of the relationship between lactation feeding patterns, litter performance, and sow characteristics on sow efficiency metrics

**DOI:** 10.1093/tas/txaf174

**Published:** 2025-12-30

**Authors:** Elly Kirwa, Beau Peterson, Caleb Grohmann, Matt Frizzo, Jeremy Perez, Ana Paula Mellagi, Rafael da Rosa Ulguim, Gustavo S. Silva

**Affiliations:** VDPAM, Iowa State University, Ames, IA, United States; Carthage Innovative Swine Solutions, LLC, Carthage, IL, United States; Carthage Innovative Swine Solutions, LLC, Carthage, IL, United States; Carthage Innovative Swine Solutions, LLC, Carthage, IL, United States; Carthage Innovative Swine Solutions, LLC, Carthage, IL, United States; Animal Medicine Departament, Federal University of Rio Grande do Sul, Porto Alegre, RS, Brazil; Animal Medicine Departament, Federal University of Rio Grande do Sul, Porto Alegre, RS, Brazil; VDPAM, Iowa State University, Ames, IA, United States

**Keywords:** Sow efficiency, wean-to-estrus interval, subsequent farrowing, subsequent total born

## Abstract

In U.S. breeding herds, data collection is widespread but often fragmented across systems. While producers rely on performance summaries, integration of these data to improve productivity remains underutilized. This study evaluated sow-level factors associated with sow efficiency, defined as weaning-to-estrus interval (WEI), percentage of sows bred within 7 days post-weaning, subsequent farrowing success, and total piglets born. Data were sourced from six lactation trials on a commercial sow farm, with sows of the same genetics (PIC line 1050), housing, and free of Porcine Reproductive and Respiratory Syndrome Virus (PRRSV) and Porcine Epidemic Diarrhea Virus (PEDV). The dataset contained 4,300 observations, including reproductive performance, daily feed intake, sow and litter weights. Generalized linear regression models were constructed with 23 variables; trial was included as a random effect, and model selection performed through manual stepwise forward selection based on biological plausibility. Pairwise comparisons were made using t-tests with the Tukey-Kramer adjustment at *P* <0.05 significance. Parity *(P* < 0.001), nursed piglets *(P* = 0.01), and average daily feed intake (ADFI0 in the first three days (*P* = 0.01) were associated with WEI. Sows nursing ≥15 piglets had a 1.3-day increase in WEI (*P* = 0.01), while ADFI <4.5kgs (10lbs) for the first three days was associated with 1-day increase in WEI (*P* <0.001). Factors associated with breeding within 7 days included parity (*P* = 0.05), first-week ADFI (*P *= 0.01), and nursed piglets (*P* = 0.009). Subsequent farrowing success was associated with prior litter size (*P = 0.02*), stillbirth rate (*P* = 0.01), first-week ADFI (*P* = 0.01), nursed piglets (*P* = 0.02), and body weight change (*P* = 0.01). Sows with ≥1 stillborn piglet had a 7% lower farrowing probability (*P* = 0.01), and those nursing >15 piglets had a 12% reduction (*P* = 0.02) in farrowing success. Factors associated with subsequent total born included parity (*P* < 0.001), previous litter size (*P* = 0.01), piglet birth weight (*P* = 0.01), caliper change *(P* = 0.04), stillbirth rate (*P 0.01*), and the interaction between body weight change and litter wean weight (*P* = 0.002). Sows with average litter birth weights <1 kg (2.4lbs) produced two more piglets than those >1.5kgs (3.5lbs). Stillbirth rates >5% reduced subsequent litter size by 2 piglets (*P* <0.05), and caliper gains >1 unit added 2 piglets compared to sows losing a unit of caliper (*P*  0.05). Overall, early lactation feed intake, litter size, and body condition were associated with reproductive outcomes. Low early lactation—first week- feed intake and high nursing burden extended WEI, delayed rebreeding, and reduced farrowing success, providing evidence-based targets to improve sow productivity.

## Teaser

Early lactation feed intake, stillbirth rate, and number of piglets suckling are associated with key reproductive outcomes in sows. These factors are linked to longer wean-to-estrus intervals, reduced farrowing success, and smaller subsequent litter, providing evidence-based targets to improve sow management and herd productivity.

## Introduction

In U.S. breeding herds, data collection is widespread; yet, too often, this information remains fragmented across different software systems. Technological advancements have expanded opportunities for data acquisition, inter-farm collaboration, and analytical approaches. Despite this potential, the practical application and integration of such data analytics within swine production systems remain underutilized (Piñeiro et al. 2019). Strategic analysis of farm-level data holds considerable promise for optimizing sow efficiency and enhancing overall herd productivity and consistency in breeding operations (Patterson and Foxcroft 2019; Koketsu and Iida 2020). Sow efficiency can be defined as wean-to-estrus interval (WEI), successful breeding within seven days post-weaning, subsequent farrowing success, and subsequent total born piglets.

Traditionally, several key production indicators commonly used at the farm level have been analyzed to evaluate sow efficiency. These metrics include annual sow productivity, typically expressed as the number of piglets weaned per sow per year (Angjelovski et al. 2014, Zhou et al. 2022); farrowing rate (Iida and Koketsu, 2018; Koketsu et al. 1997); WEI (Poleze et al. 2006, Gianluppi et al. 2020); pre-weaning mortality (Nuntapaitoon and Tummaruk 2018; Will et al. 2024) and sow prolificacy, often assessed by the total number of piglets born and the number born alive per litter (Tani et al. 2018). These indicators remain useful for monitoring specific stages of reproduction and identifying short-term changes in performance. However, because each metric reflects only a single component of the production cycle, they provide a fragmented view of sow efficiency and do not fully capture a sow’s cumulative contribution across multiple parities. This limitation underscores the need for more holistic measures of sow efficiency, which is the focus of the present study.

Lactation represents one of the most metabolically demanding phases in the reproductive cycle of mammals, and this is especially true for sows (Tokach et al. 2019). Advancements in genetics have significantly increased litter sizes, a trend that remains a primary focus in swine breeding strategies (Baxter et al. 2013; Silalahi et al. 2016). This elevated requirement may predispose sows to a pronounced negative energy balance during lactation, which can adversely affect follicular development (Costermans et al. 2020), result in longer WEI and lower farrowing rates, impacting their subsequent and lifetime productive performance. Despite the increased demand during lactation, sows often fail to achieve their nutritional needs (Verstegen et al. 1985). To compensate, sows mobilize their body energy reserves to maintain milk output (Eissen et al. 2000; Strathe et al. 2017). While this physiological adaptation supports short-term productivity, excessive depletion of body tissues may impair reproductive performance in subsequent cycles, including an extended WEI (Lundgren et al. 2014; ­Thekkoot et al. 2016).

Extensive research has demonstrated that inadequate nutrient intake during lactation compromises sow reproductive efficiency and diminishes overall productivity (Koketsu et al. 1997; de Jong et al. 2013; Kim et al. 2013; Ampode et al. 2023; Rodríguez et al. 2023; Estrada et al. 2024). However, limited research has comprehensively integrated individual sow-level characteristics and litter performance metrics to determine key indicators of sow efficiency defined by more than one outcome. Therefore, the objective of the study was to assess the relationship between lactation feeding patterns along with litter performance, and sow characteristics on sow efficiency metrics defined as WEI, percentage of sows bred within 7 days post-weaning, subsequent farrowing success, and subsequent total piglets born.

## Materials and methods

### Overview

**Fig. 1.**
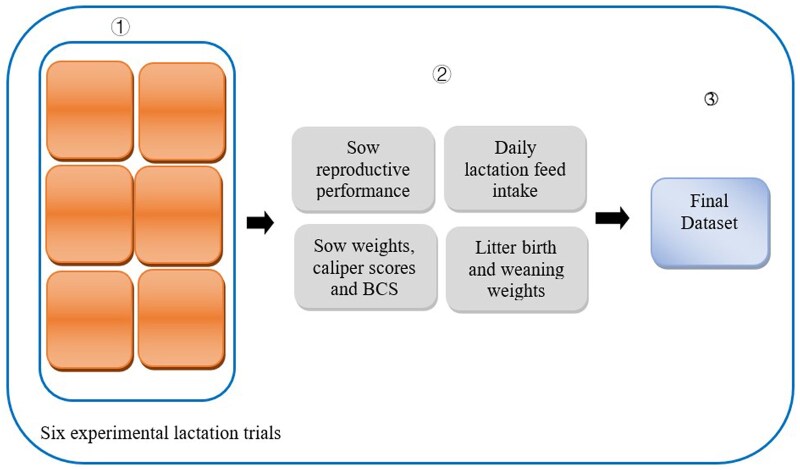
Flow diagram describing the different data sources used to build the final dataset. (1) The six lactation trials; (2) Variables from different data streams, including key performance indicators, lactation feeding, sow and litter characteristics; (3) Final dataset containing potential risk factors and outcomes in every trial.

This observational retrospective study utilized individual-level data from six independent lactation trials conducted between August 2021 and July 2022 on a commercial sow farm in the Carthage System (Carthage, IL, USA) in the Midwestern United States. Trial-specific variables were aggregated into a single dataset from 3 different sources, including sow performance, litter characteristics, and daily lactation feed intake. Figure 1 illustrates the various data sources used to construct the final dataset. A single master file from each trial was then combined to form the final dataset used in the analysis to evaluate sow-level risk factors associated with reproductive efficiency, and subsequent performance.

### Study design and selection criteria

All trials were conducted on the same farm and under standardized management and housing conditions. Sows were of the same genetic line (PIC 1050; PIC, Hendersonville, TN) and were negative for porcine reproductive and respiratory syndrome virus (PRRSV) and porcine epidemic diarrhea virus (PEDV) during the study period. The study population included sows with complete lactation and reproductive data. The final aggregated dataset consisted of 4,300 sows. Sows of parity ≥8 were excluded due to potential age-related confounding and to maintain balanced parity distributions.

### Source of data

The study observations included reproductive performance, daily feed intake during lactation, pre- and post-farrowing sow body weights, caliper measurements, litter birth weight and weaning weight, and subsequent reproductive performance. Caliper measurement is a body-condition assessment tool used to measure changes in sow backfat or overall body condition in sows. It provides a standardized, objective estimate of tissue reserves and energy balance, helping identify whether a sow is losing or maintaining condition across the lactation period. Sow body weights were measured upon entry into the farrowing room at 112 days of gestation and again at weaning, using a calibrated scale (Digistar-SW300, DigiStar LLC, Fort Atkinson, WI). Adjusted sow body weight at farrowing was calculated by subtracting estimated *in-utero* litter weight, as described by Thomas et al. (2016). Sows had ad libitum access to feed from farrowing through weaning. Total lactation intake was determined by hand-delivering feed using a calibrated scoop. Lactation length was recorded to compute average daily feed intake (ADFI). Daily feed refusals were weighed and removed from the total lactation intake. Nursed piglets were defined as the total number of piglets suckling at the start of the study and throughout lactation period. The total number of pigs weaned from each sow was calculated by subtracting the number of falloffs and mortalities from the starting litter (ie nursed piglets). Litter birth weight and weaning weight were obtained either by summing individual piglet weights using a tared empty tub on a scale or by weighing the entire litter at once (UWE electronic scale, model AMP-150). Piglet cross-fostering was performed within 24 hours of farrowing as needed according to the sow’s teat count. Additionally, various binning approaches were explored for the numerical variables, including quantiles for lactation length and litter size, with consideration of the distribution of each variable in the dataset. Table A in the supplementary material provides a detailed description of each variable, including its definition, type, and categories (or levels).

### Statistical analysis

#### Regression models and outcomes

A total of 23 variables were evaluated in each model, and trial ID was included as a random effect to account for potential variations across different trials. All regression models were built using the R program. Mixed logistic regression models assessed the factors associated with the percentage of sows bred within 7 days post-weaning and subsequent farrowing success. A Poisson mixed regression model was used to assess factors associated with WEI (days), while a linear mixed regression model assessed factors associated with subsequent total born piglets. The model-building process across the outcomes involved a manual stepwise forward selection approach, where interactions and confounders were tested based on biological relevance. Pairwise comparisons were tested using t-tests with the Tukey-Kramer adjustment, considering *P*-values <0.05 as statistically significant.

### Univariate analysis

Univariate analysis was performed on all 23 explanatory variables to determine their association with the four outcomes independently. Variables that yielded a *P*-value of ≤0.20 were considered for inclusion in the subsequent multivariable modeling (Dohoo et al. 2009). Prior to multivariable analysis, the explanatory variables were assessed for pairwise correlations using Spearman’s rank correlation to detect potential multicollinearity. Variables were considered collinear if the correlation coefficient exceeded 0.70.

### Multivariable analysis

The multivariable model-building process involved a manual stepwise forward selection approach (Dohoo et al. 2009). Variables that showed potential associations in the univariate analysis (*P ≤* 0.20) were sequentially added to the model based on their statistical significance and biological plausibility. At each step, the contribution of the variable to the model was assessed using likelihood ratio tests, and only those variables that significantly improved model fit (*P <* 0.05) were retained. Potential confounding variables were evaluated by examining changes in the estimated coefficients of primary explanatory variables upon their inclusion. A variable was considered a confounder if its inclusion altered the coefficient of another variable by more than 20%. Biologically plausible interaction terms and pairwise comparisons were tested using t-tests with Tukey-Kramer adjustment, considering *P*-values <0.05 as statistically significant.

## Results

### Overview

Table 1 describes the summary statistics of sow and litter characteristics by parity. The final aggregated dataset consisted of 1,044 parity 1, 880 parity 2, 1,523 parity 3 to 5, and 853 parity 6 + sows. Mean sow weights and lactation ADFI increased with parity. Conversely, WEI decreased with parity. While the number of piglets born alive was consistent across parities, the stillbirth rate increased with parity, rising from 5.7% in Parity 1 to 11.8% in Parity 6 + sows. A summary of the main factors associated with the different key reproductive performance metrics in univariate and multivariable analysis is presented in Tables 2 and 3.

**Table 1.** Descriptive statistics (mean and standard deviation) of key sow performance indicators by parity.

Outcomes		Parity 1 (*n* = 1044)	Parity 2 (*n* = 880)	Parity 3–5 (*n* = 1523)	Parity 6+ (*n* = 853
**Born Alive, n**	12 (2.9)		11 (3.3)	14 (2.4)	14 (3.6)
**Stillbirth rate, %**	5.7 (4.6)		5.5 (3.5)	8.6 (5.1)	11.8 (6.2)
**Subsequent Total Born, n**	15 (3.9)		16 (4.2)	17 (4.6)	15 (3.8)
**WEI, days**	6.4 (5.6)		6.0 (5.1)	5.0 (3.9)	4.7 (3.3)
**Lactation ADFI^1^, kgs/day**	5.7 (1.5)		7 (1.1)	7.9 (1.1)	7.9 (1.1)
**Sow weight on entry to farrow, kgs**	202 (20)		223.6 (21.2)	249 (21.9)	264.4 (20.5)
**Sow weight at weaning, kgs**	170 (17.8)		195 (20)	228 (22.5)	250.4 (20.7)

1 ADFI = Average Daily Feed Intake.

**Table 2.** Results from univariate analysis of risk factors associated with wean-to-estrus interval (WEI, days), percentage of sows bred within 7 days post-weaning, subsequent farrowing likelihood, and subsequent total born piglets.

Outcome	Risk factor	*P*-value
**Wean–to–Estrus Interval (WEI), days**	Parity	<0.001
	Sow body weight change	<0.001
	Litter size	0.01
	Caliper change	<0.001
	Caliper at weaning	*<*0.001
	ADFI First 3 days of lactation	0.01
	ADFI First 7 days of lactation	0.08
	Nursed litter	0.04
	Pigs weaned	0.13
	Litter wean weight	0.01
	Season	0.3
	Stillborn	0.04
	Feed refusal events	0.02
	Energy intake	0.15
	Lactation length	0.04
**Percentage of sows bred within 7 days post-weaning, %**	Parity	<0.001
	Caliper change	<0.001
	Nursed litter	0.01
	ADFI First 3 days of lactation	0.13
	ADFI First 7 days of lactation	0.13
	Farrowing season	0.08
	Caliper at weaning	0.02
	Sow body weight change	0.14
	Litter wean weight	0.03
	Litter size	0.22
	Treatment during lactation	0.16
**Subsequent farrowing success (yes/no)**	Parity	0.01
	Farrowing season	0.13
	Stillbirth rate	0.03
	Piglets nursed	0.06
	Litter size	0.03
	Caliper at weaning	0.07
	Sow body weight change	0.08
	Lactation length	0.10
	Caliper change	0.25
	ADFI First 3 days of lactation	0.12
	ADFI First 7 days of lactation	0.03
**Subsequent total born piglets, n**	Parity	<0.001
	Previous litter size	<0.001
	Caliper change	<0.001
	ADFI First 3 days of lactation	0.12
	ADFI First 7 days of lactation	0.06
	Piglets nursed	0.10
	Feed refusal events	0.04
	Energy intake	0.15
	Sow body weight change	0.03
	Lactation length	0.08
	Stillborn rate	0.06
	Caliper at weaning	0.26

* Feed refusal events represent the number of lactation days in which a sow showed no voluntary feed intake during one or more scheduled feeding periods, as recorded by the feeding system.

**Table 3.** Risk factors associated with wean-to-estrus interval (WEI, days), percentage of sows bred within 7 days post-weaning, subsequent farrowing likelihood, and subsequent total born.

Outcomes	Risk factors	Categories (levels)	Least Squares Means*	*P*-value
**WEI (days)**	Parity	Parity 1	7.73^a^	<0.001
		Parity 2	6.96^a^	
		Parity 3–5	5.03^b^	
		Parity 6+	4.72^b^	
	Piglets nursed	<12 piglets	5.25^a^	0.01
		12–14 piglets	5.81^b^	
		≥15 piglets	6.30^c^	
	ADFI first 3 days	Low: <4.5kgs	6.64^a^	0.01
		Medium: 4.6–6.3kgs	6.08^b^	
		High: ≥6.3kgs	5.79^b^	
**Percentage of sows bred within 7 days post-weaning**	Parity	Parity 1	85.6%^a^	0.05
		Parity 2	88.5%^a^	
		Parity 3–5	93.6%^b^	
		Parity 6+	95.6%^b^	
	ADFI during the first week of lactation	Low: <4.5kgs	78.3%^a^	0.01
		Medium: 4.6–6.3kgs	83.4^ab^	
		High: ≥6.3kgs	87.7%^b^	
	Piglets nursed	<12 piglets	94.3%^b^	0.009
		12–14 piglets	91.7%^b^	
		≥15 piglets	88.0%^a^	
**Subsequent farrowing success**	Stillborn rate	At least 1 stillborn	76.2%^a^	0.01
		No Stillborn	83.1%^b^	
	ADFI first 7 days	Low: <4.5kgs	77.3%^a^	0.01
		Medium: 4.6–6.3kgs	81.6%^a^	
		High: ≥6.3kgs	82.9%^b^	
	Sow Body weight change	Lost	80.3%^a^	0.01
		Gain	85.6%^b^	
	Piglets nursed	<12 piglets	85.2%^b^	0.02
		12–14 piglets	78.9%^ab^	
		≥15 piglets	73.7%^a^	
**Subsequent total born piglets**	Parity	Parity 1	14.8^9a^	<0.001
		Parity 2	15.91^ab^	
		Parity 3–5	16.26^b^	
		Parity 6+	15.23^ab^	
	Previous litter size	<9 piglets	14.92^a^	0.01
		10–14 piglets	15.32^a^	
		≥15 piglets	16.78^b^	
	Piglet birth weight	< 1.0 kg	16.43^a^	0.016
		1.1–1.5kgs	15.42^b^	
		>1.5kgs	14.86^ab^	
	Sow caliper change	Loss	14.22^b^	0.04
		No Change	15.43^ab^	
		Gain	16.13^a^	
	Stillbirth rate, %	<5% stillbirth rate	15.82^a^	0.01
		≥5% stillbirth rate	13.74^b^	
	Interaction between sow body weight change and litter wean weight			0.002
	Gain	Medium 5.0–6.8 kgs	16.06^c^	
	Gain	Light <4.9 kgs	15.56^abc^	
	Gain	Heavy >6.8 kgs	14.91^ab^	
	Lost	Medium 5.0–6.8 kgs	15.08^b^	
	Lost	Light <4.9 kgs	15.74^abc^	
	Lost	Heavy >6.8kgs	16.05^ac^	

* Superscript letters mean a statistical significance at *P* < 0.50 between levels within factors.

* Sow body weight change calculated as the difference of body weight on entry to farrow, adjusting for in-utero piglets and weight at weaning.

* Litter wean weight is the average weight of the litter at weaning.

a,b,c The letters above the bars indicate significant differences among groups (*P* < 0.50).

### Results from univariate analysis

Table 2 shows results from univariate analysis, with a *P*-value <0.2 considered significant. Overall, parity, sow body condition (caliper change, body weight change), and ADFI during lactation were consistently associated with WEI, percentage of sows bred within 7 days post-partum, subsequent farrowing success, and total piglets born. Litter characteristics, including litter size, piglets weaned, and stillbirths, also showed significant associations with WEI, subsequent farrowing success, and total piglets born. These findings indicate that both sow-level and litter-level factors contribute to variation in subsequent reproductive performance after adjusting for other variables in the model.

### Results from multivariable analysis

**Fig. 2.**
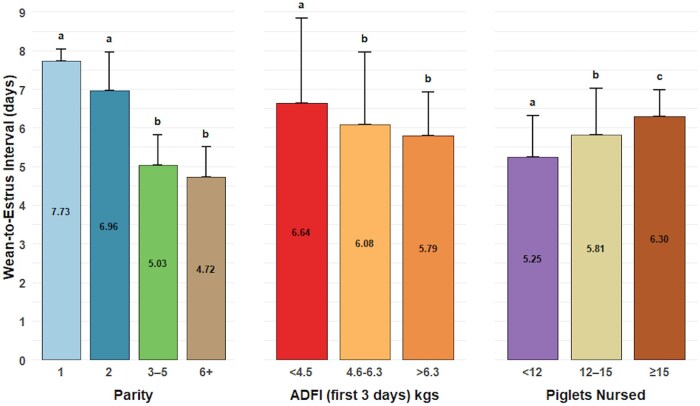
Risk factors associated with wean-to-estrus interval (WEI). the plot presents the effects of parity, lactation average daily feed intake (ADFI) first 3 days post-farrowing, and nursed pigs on WEI (in days). the bars represent the mean WEI for each category, with error bars indicating the 95% upper confidence intervals. The letters above the bars indicate significant differences among groups (*P* < 0.05).

Factors associated with WEI shown in Fig. 2 included parity (*P <* 0.001), nursed piglets (*P = * 0.01), and ADFI in the first three days of lactation (*P = * 0.01), which was confounded by farrowing (*P = * 0.03). Sows with more than 15 piglets nursed during lactation had a 1.3-day increase in WEI compared to those that nursed fewer than 12 piglets. Lactation ADFI less than 4.5kgs (10lbs) in the first 3 days was associated with a 1-day increase in WEI compared to >4.5kgs (10lbs) of lactation ADFI.

**Fig. 3.**
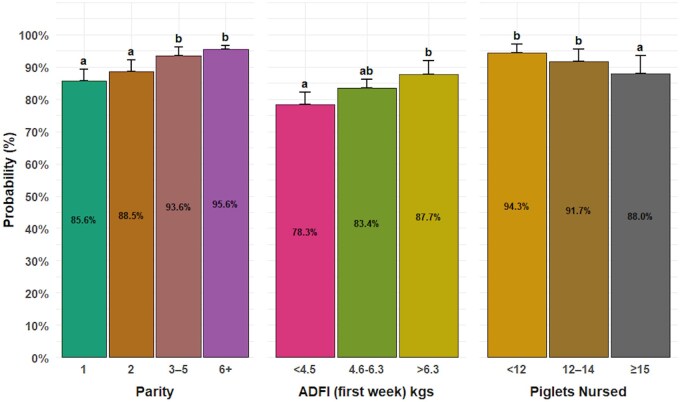
Factors associated with the percentage of sows bred within 7 days post-weaning. The plot presents the effects of parity, average daily feed intake (ADFI) in the first week of lactation, and the number of nursed pigs. The bars represent the mean probability for each category, with error bars indicating the 95% upper confidence intervals. The letters above the bars indicate significant differences among groups (*P* < 0.05).

The percentage of sows bred within 7 days post-weaning was associated with parity (*P = *0.05), number of nursed piglets (*P = *0.009), and ADFI in the first week of lactation (*P = *0.01) as indicated in Fig. 3. Sows nursing more than 15 piglets and those with lactation ADFI less than 4.5kgs (10lbs) in the first week had a 3.7% and 9% decrease in probability of being bred within 7 days post-weaning, compared to sows nursing less than 12 piglets and lactation ADFI >4.5kgs (10lbs) in the first week, respectively.

**Fig. 4.**
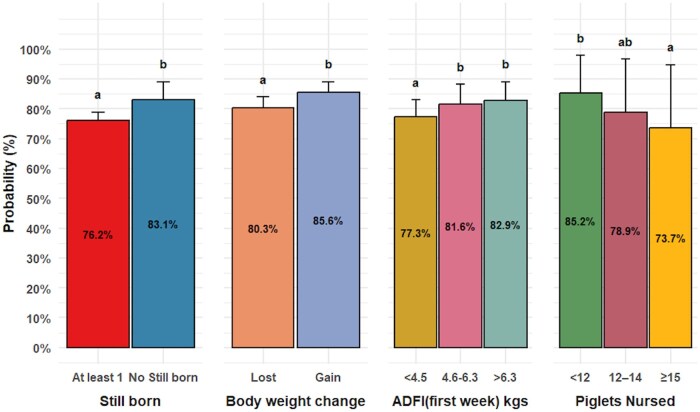
Factors associated with subsequent farrowing success. The plot presents the effects of stillborn, lactation average daily feed intake (ADFI) first 7 days, piglets after cross-fostering, and body weight change on subsequent farrowing rate. The bars represent the mean subsequent farrowing rate for each category, with error bars indicating the 95% upper confidence intervals. The letters above the bars indicate significant differences among groups (*P <* 0.05).

For subsequent farrowing success, significant factors included previous piglets nursed (*P = *0.02), stillborn (*P = *0.01), ADFI during the first week of lactation *(P = *0.01), and body weight change (*P = *0.01) as shown in Fig. 4. Sows with at least one stillborn piglet showed a 7% lower probability of farrowing in the subsequent cycle, while those with more than 15 nursed pigs had a 12% decrease in farrowing likelihood.

**Fig. 5.**
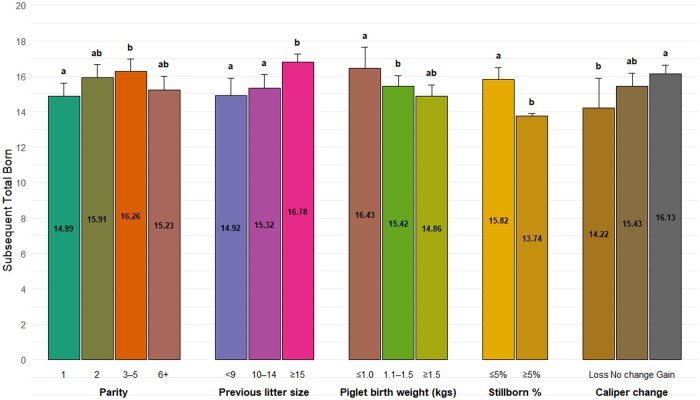
Factors associated with subsequent total born. The plot presents the effects of parity, previous litter size, piglet birth weight, percentage of stillborn, and sow caliper change on subsequent litter (total born). the bars represent the total piglets born in subsequent farrowing for each category, with error bars indicating the 95% upper confidence intervals. The letters above the bars indicate significant differences among groups (*P <* 0.05).

Factors associated with subsequent total born included parity (*P <*0.001), previous litter size (*P = *0.01), piglet birthweight (*P = *0.01), caliper change (*P = *0.04), still birth rate (*P = *0.01), as shown in Fig. 5. Additionally, there was significant interaction between sow body weight change and litter wean weight (*P = *0.002), as shown in Table 3. Sows that previously farrowed more than 14 piglets had on average, one additional piglet in the subsequent litter compared to those that farrowed <9 piglets, whereas sows with lower average litter birth weights (<1 kg(2.4lbs)) produced two more piglets compared to higher birth weights (>1.5kgs(3.5lbs)). More than 5% stillbirth rate was associated with a decrease in 2 pigs in subsequent farrowing (*P <*0.05). Additionally, sows that gained at least 1 unit of caliper during lactation had 2 more piglets in the subsequent litter compared to those that lost 1 unit of caliper (*P <* 0.05).

## Discussion

This study leveraged integrated sow-level data throughout the lactation period, combining metrics such as feed intake, body weight changes, caliper, and litter performance. The integration of these diverse yet biologically interrelated data streams enabled a more comprehensive understanding of sow productivity and reproductive efficiency. The combination of these variables provided an opportunity to explore inter-relationships that are often overlooked when variables are assessed in isolation. Specifically, the study examined the influence of sow feed intake and body condition during lactation on subsequent litter outcomes, offering insights into actionable biological and ­management factors.

WEI is a critical measure of reproductive performance, closely tied to sow physiological recovery post-lactation, timely rebreeding, and overall efficiency in sows. (Koketsu et al. 1997; Poleze et al. 2006; Koketsu and Iida 2017; Ordaz et al. 2024). Typically, sows exhibit estrus within 7 days following weaning (Almeida et al. 2020; Lopes et al. 2020; Carrión-López et al. 2022; Reeb and Kellner 2022; Di Muzio et al. 2023; Hilgemberg et al. 2024).

Our findings indicate that parity, number of nursed piglets, and ADFI during the first three and seven days of lactation were significantly associated with the WEI and the likelihood of successful breeding within seven days post-weaning. Specifically, sows nursing more than 15 piglets exhibited an average increase of 1.3 days in WEI, while those with an ADFI below 4.5 kgs (10 lbs) during the first three days of lactation experienced an approximate one-day extension in WEI. This is explained by higher lactational demand and increased suckling intensity that suppresses hypothalamic-pituitary-ovarian axis activity, thereby delaying post-weaning reproductive recovery. These findings are consistent with previous studies, which have shown that a number of piglets suckling delays estrus resumption (Knox and Zas 2001; Koketsu et al. 2017; Nam and Sukon 2020) and subsequent farrowing rate (Tokach et al. 2019). Additionally, Quesnel et al. 2007 and Decaluwé 2013 showed that inadequate early lactation nutrition compromises metabolic signals required for timely estrus resumption due to negative energy balance and reduced luteinizing hormone pulsatility. Longer WEI in first- and second-parity sows, as compared to older parities, is consistent with previous studies (Koketsu et al. 2017; Dimitrov et al. 2018; Segura-Correa et al. 2013), and is attributed to the physiological demands and delayed recovery of reproductive function postpartum observed in primiparous sows (Ordaz-Ochoa et al. 2013).

The presence of stillborn piglets impaired both farrowing success and subsequent litter size. Biologically, stillbirth reflects intrapartum complications or delayed uterine recovery, which impair reproductive tract function (Björkman et al. 2022). Similarly, large litters impose high metabolic requirements on sows, increasing the risk of negative energy balance and ­compromising follicular development and oocyte quality ­(Costermans et al. 2020). These physiological stressors disrupt the hormonal milieu necessary for timely estrus and successful conception, thereby reducing the likelihood of subsequent farrowing.

Previous studies have reported that prolonged farrowing and long inter-piglet birth intervals are major contributors to stillbirth and are linked to reduced subsequent fertility, including higher repeat breeding rates after weaning (Adi et al. 2022; 2022; Van den Bosch et al. 2022; Schoos et al. 2023; Ngo et al. 2024). Additionally, sows with low feed intake during lactation, weaned lighter and fewer piglets, and have fewer piglets born in the next farrowing, along with prolonged WEI (Estrada et al. 2024). Furthermore, Arend et al. (2023), in a controlled study of primiparous sows, found that nursing large litters lost more body condition, but there was no negative impact on subsequent farrowing rate.

Parity remains a well-established determinant of litter size and reproductive performance. Sows in mid-parity (3 to 5) typically achieve peak litter output, whereas both primiparous and older sows often exhibit reduced prolificacy and increased stillbirth rates due to physiological immaturity or reproductive senescence, respectively (Quiniou et al. 2002; Koketsu et al. 2017; Buthelezi 2024; Thiengpimol et al. 2024). In the current study, sows that previously farrowed more than 14 piglets had, on average, one additional piglet in the subsequent litter, whereas those with lower birth weights produced two more piglets in subsequent farrowing. Prior litter size has been reported as a robust predictor of future reproductive potential (Boonkum et al. 2025). However, increases in total born are frequently accompanied by lower mean birth weight and greater within-litter weight variation, both of which adversely impact neonatal viability (Milligan et al. 2002; Yuan et al. 2015). Piglet birth weight and uniformity are critical predictors of survival and overall litter success, with lighter piglets facing significantly higher mortality risk (Mbuthia et al. 2025; Harper and Bunter 2024). The average birth weight of piglets decreases as litter size increases, which in turn antagonizes reproductive performance (Knap 2023). However, breeding strategies that balance litter size and birth weight successfully neutralize this trade-off, improving both birth weight and subsequent piglet survival over time.

In the current study, sows that gained at least one unit of caliper during lactation had two more piglets in the subsequent litter compared to those that lost one unit of caliper. Similarly, Sens Júnior et al. (2023) reported an average stillbirth rate of 5% and linked this rate to farrowing duration and litter size, highlighting the ongoing risks associated with stillbirths in sows, which can reduce subsequent performance. Bayesian modeling by Teixeira Costa et al. (2024) showed that older sows with ≥15% stillborn in one litter had a 2.5-times higher risk of stillbirth in subsequent farrowing, implying a measurable loss in reproductive output. Additionally, body condition dynamics, as reflected by sow weight and caliper measurements, are essential for optimizing reproductive efficiency. Caliper change, a proxy for backfat and body condition, follows a curvilinear relationship with reproductive success, where both under- and over-conditioned sows exhibit suboptimal outcomes during lactation (Koketsu et al. 2017). The results in the current study corroborate findings of (Authement and Knauer 2023), who demonstrated that each one-unit increase in caliper score during lactation was associated with approximately two extra piglets born alive in the subsequent litter. Mellagi et al. (2013) indicated that higher weight loss was not a risk factor in parity 3–5 sows; however, it decreased subsequent litter size.

The observed interaction between sow body weight change and litter weaning weight for subsequent litter size highlights the role of metabolic resilience in reproductive success. Sows capable of weaning heavier litters without excessive weight loss tend to return to estrus more promptly and maintain higher fertility, reflecting efficient energy use and feed intake. Correspondingly, significant body weight loss is associated with delayed estrus and reduced conception, due to impaired metabolic and hormonal function (Hu et al. 2019; Costermans et al. 2023). Therefore, selecting sows that can sustain a large litter while maintaining a healthy body condition to improve lifetime productivity is essential (Peltoniemi et al. 2021; Bortolozzo et al. 2023).

This study has limitations. First, while the use of detailed, sow-level data, including daily lactation feed intake, sow and litter weights, enhanced the granularity and biological relevance of our analysis, such variables are not routinely recorded in most commercial production systems, potentially limiting the external validity. Additionally, the study was conducted within a single production system with uniform genetics, free of PRRSV and PEDV, limiting its generalizability to other herds with differing practices and health statuses. Future research involving diverse commercial settings and broader datasets is warranted to strengthen and extend these findings.

## Conclusion

These findings provide actionable insights for optimizing sow management, enabling targeted lactation feeding management and reproductive strategies to improve farrowing rates and overall herd productivity. Enhancing sow reproductive efficiency requires prioritizing early lactation feed intake, with a target of at least 4.5kgs (10lbs)/day during the first week postpartum. Monitoring cross-fostering is essential to prevent large suckling litters on sows, which can compromise WEI and farrowing success.

## Supplementary Material

txaf174_Supplementary_Data

## List of Abbreviations:

WEI: Wean to estrus interval
ADFI: Average daily lactation feed intake
PRRSV: Porcine Reproductive and Respiratory Syndrome virus
PEDV: Porcine Epidemic Diarrhea virus
PIC: Pig Improvement Company
IL: Illinois
TN: Tennessee
WI: Wisconsin
α: Probability of type I error
CI: confidence interval
