# Supplementary material for: Assessment of the relationship between lactation feeding patterns, litter performance, and sow characteristics on sow efficiency metrics
Source: Transl Anim Sci. 2025 Dec 30;10:txaf174. doi: 10.1093/tas/txaf174 (PMC12914738; doi:10.1093/tas/txaf174)
Supplement: txaf174_Supplementary_Data [file txaf174_supplementary_data.docx]

**Table A**. Description of variables included in the analysis of sow efficiency

| Variable name | Definition | Variable type | Categories / Levels |  |
| --- | --- | --- | --- | --- |
| Trial ID | An identifier for each independent lactation trial | Categorical (random effect) | Trial 1–6 |  |
| Sow ID | Unique identifier for each sow | Categorical | Unique per sow |  |
| Parity | Number of farrowings at the time of observation | Categorical | Parity 1; Parity 2; Parity 3–5; Parity ≥6 |  |
| Lactation length (days) | Number of days from farrowing to weaning | Continuous | Also explored as quantiles |  |
| Adjusted sow body weight at farrowing (kg) | Sow body weight at entry to farrowing room minus estimated in-utero litter weight | Continuous |  |  |
| Sow body weight at weaning (kg) | Sow body weight measured at weaning | Continuous |  |  |
| Sow body weight change (kg) | Difference between weaning and adjusted farrowing body weight | Categorical | Loss; No change; Gain |  |
| Caliper score at farrowing | Body condition score measured using caliper at farrowing | Categorical | Thin (5-8), Ideal(9-12), Fat(>13) |  |
| Caliper score at weaning | Body condition score measured using caliper at weaning | Categorical | Thin (5-8), Ideal(9-12), Fat(>13) |  |
| Caliper change | Difference between weaning and farrowing caliper scores | Continuous | Lost >1 unit;  No change 0;  Gain +1unit |  |
| Average daily feed intake (ADFI), total lactation (kg/day) | Total lactation intake divided by lactation length | Continuous |  |  |
| ADFI first 3 days (kg/day) | Average daily feed intake during the first 3 days of lactation | Categorical | Low< 4.5 kg;  Medium 4.6-6.3kg  High ≥ 6.3 kg |  |
| ADFI first 7 days (kg/day) | Average daily feed intake during the first 7 days of lactation | Continuous | Low< 4.5 kg;  Medium 4.6-6.3kg  High ≥ 6.3 kg |  |
| Number of feed refusals | Number of days during lactation with recorded feed refusal events | Count / continuous |  |  |
| Piglets nursed | Number of piglets suckling throughout the lactation period | Categorical | < 12 piglets; 12–15piglets; > 15 piglets |  |
| Piglets weaned | Number of piglets weaned per sow | Continuous /  Categorical | <10 piglets; 10-14 piglets; > 14piglets |  |
| Total piglets born  (Previous litter size) | Total number of piglets born per litter | Continuous | < 9piglets; 9–14piglets ; > 14piglets | |
| Stillborn | Presence or absence of still born in a litter | Categorical | At least 1 still born;  No still born | |
| Stillbirth rate (%) | Proportion of stillborn piglets relative to total born | Continuous | ≤ 5% Stillbirth rate;  > 5% Stillbirth rate | |
| Litter birth weight (kg) | Total weight of all piglets at birth | Categorical | < 1.0kg  1.1-1.5kgs  >1.5kgs | |
| Average piglet birth weight (kg) | Litter birth weight divided by number of piglets born | Continuous | < 1.0; 1.0–1.5; > 1.5(kgs) | |
| Litter weaning weight (kg) | Total weight of all piglets at weaning | Categorical | Quantiles | |
| Wean-to-estrus interval (WEI, days) | Number of days from weaning to first observed estrus | Count |  | |
| Bred within 7 days post-weaning | An indicator of whether the sow was bred within 7 days after weaning | Binary | Yes; No | |
| Subsequent farrowing success | An indicator of whether sow farrowed in the subsequent cycle | Binary | Yes; No | |
| Subsequent total piglets born | Total number of piglets born in the next farrowing | Continuous |  | |
